# Eco-friendly sustainable farming: Enhancing summer tomato (*Lycopersicon esculentum* mill.) yield with jute non-woven agro textile Mulch

**DOI:** 10.1016/j.heliyon.2025.e42039

**Published:** 2025-01-16

**Authors:** Nilimesh Mridha, Dipak Nayak, Ashok Yadav, Tilak Mondal, Rakesh Kr Ghosh, Manik Bhowmick, Atul Singha, D.P. Ray, B.S. Manjunatha, Avijit Das, D.B. Shakyawar, Sourav Paul, Amit Das, Santanu Mukherjee, Ravinder Kumar

**Affiliations:** a ICAR-National Institute of Natural Fibre Engineering and Technology, Kolkata, 700040, India; b ICAR-Central Institute of Sub-tropical Horticulture (RRS), Malda, 732101, India; c ICAR-Central Agroforestry Research Institute (ICAR-CAFRI), Jhansi, UP, 284003, India; d ICAR- National Bureau of Agricultural Insect Resources, Bengaluru, 560 024, India; e School of Agriculture, Shoolini University of Biotechnology and Management Sciences, Solan, 173229, India; f Division of Plant Pathology, ICAR-Indian Agricultural Research Institute, New Delhi, 110012, India

**Keywords:** Agro textile, Jute non-woven, Biodegradable, Summer tomato, Fruit quality, Soil parameters

## Abstract

Jute-based non-woven was engineered using 50 % pure jute with 50 % caddis (mill waste of jute) on a weight basis. Jute non-woven mulch with 100 % jute (100:0) of 250, 450, and 650 g per square meter (GSM), the blend non-woven of pure jute with caddis (50:50) of 250, 450, and 650 GSM, plastic (50 μm) and rice straw mulch were evaluated on summer tomato crop at the ICAR-CISH regional station in Malda, West Bengal, India. Jute non-woven fabrics of higher thickness with lower water flow and transmissivity increase soil moisture content over no mulch (∼70 %) and plastic mulch (∼9.3 %). The temperature throughout the soil depths for all mulch treatments was lower over no mulch, and for 650 GSM, it was the lowest. Various soil parameters, plant growth parameters, microbial growth, nutrient uptake, and water use efficiency (WUE) under jute non-woven have been improved significantly over no mulch and also over plastic mulch. Jute mulch of 650 GSM, 100:0, followed by 250 GSM, 50:50, and 450 GSM, 100:0, outperformed other mulching materials for tomato yield and fruit quality parameters. The yield of summer tomato crops under jute non-woven (650 GSM, 100:0) has increased significantly by 84 % over no mulch and 28 % over plastic mulch. Therefore, jute non-woven mulch can be a potential alternative to plastic mulch for eco-friendly and sustainable tomato production.

## Graphical abstract

**Figure:**
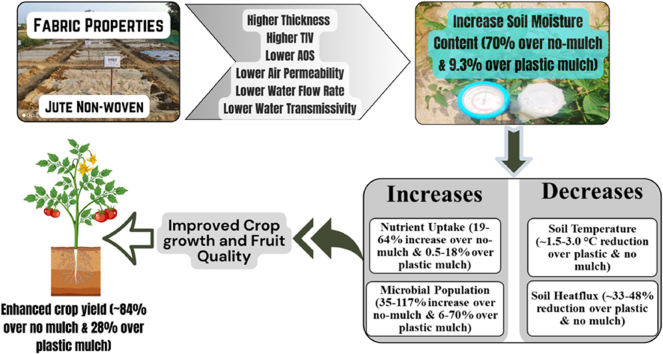

## 1 Introduction

Mulching is a beneficial agricultural technique that manipulates the environment for growing crops leading to higher yields and better-quality products by modulating soil temperature, preserving moisture levels, and minimizing soil moisture evaporation [1]**.** Plastic mulching in agricultural soil is a common practice to enhance crop productivity by modifying soil conditions. While plastic mulches offer benefits such as moisture conservation, weed control, and regulation of soil temperature for improved crop growth, the prolonged use of plastic mulch has been associated with the introduction of microplastics into the soil, which can have long-term impacts on soil health and ecosystem dynamics [2], soil structural loss, and reduced activity of soil microorganisms [3]**.** In 2018, 360 million tons of plastic mulch were produced globally, with Asia contributing to 51 % of the production [4], increasing each year [5]. In China alone, the largest user of plastic mulch films, around 14.7 × 10^5 tons of plastic waste are generated annually for disposal [6]. The widespread adoption of polyethylene (PE) mulch films in agriculture has led to a notable accumulation of plastic waste in soil ecosystems [7]. The long-term application of plastic film mulching can adversely affect crop growth and soil quality due to the persistence of plastic residues and the accumulation of microplastics in the soil [8]. Several research studies have shown that prolonged, plastic mulch application increases microplastic pollution in agricultural soils, emphasizing the need to evaluate further the ecological risks associated with microplastics in soil ecosystems [9]. Several studies showed crop yields decreased when residual plastic film was left in the soil.

Plastic pollution, known as 'white pollution,' is today's biggest concern due to its comparable and undesirable environmental impacts. Plastic fragments have been identified as capable of absorbing persistent toxins in the environment, leading to disruptions in both terrestrial and aquatic ecosystems [10,11]. The disposal phase of synthetic materials in their life cycle is particularly damaging to the environment, resulting in significant direct economic and social costs [12,13]. Plastic waste management is complex due to its intricate structure, which results in harmful emissions and residual ash [14]. Plastic contamination with other waste types also often limits recyclability [15]. Organic mulches such as straw, husks, and reeds have their limitations of nutrient tie-up as these mulches can compete with crops for nutrients during the decomposition process, leading to temporary nutrient deficiencies in the soil and affecting plant growth and yield [16] and may not provide sufficient weed control, leading to weed competition with crops and reducing overall yields [17]. Among biodegradable mulch materials, jute-based non-woven agrotextiles play a significant role and can offer sustainable alternatives for crop production over synthetic agrotextiles mulch. They can withstand solar and UV radiation much better than synthetic fibers. They can retain moisture for extended periods [1]. The agrotextile mulch prepared from jute fibre crops which acts as a significant net CO_2_ sink [18] could provide renewable and eco-friendly solutions to the plastic menace. The crop is cultivated over an area of 1.54 M ha with a production of 3.6 M tones globally [19].

India has the highest jute growing area of 0.76 M ha with an average fibre yield of 2.55 tonnes per ha and a total production of 1.9 M tones [20]. The production of jute-based non-woven materials aligns with sustainable development goals and eco-conscious practices. Jute fibers, being biodegradable and renewable, offer an environmentally friendly alternative for various applications, including agrotextiles, packaging, and composite materials [21]. Jute fibres have good potential as non-woven mulch due to their unique properties such as air permeability, water permeability & transmissivity, thermal, and compressional properties, etc. [22]. The jute industry generates about 40,000 tonnes of processing waste of unspinnable short jute fibre as a by-product, commonly known as caddis [23]. This caddis, considered waste, can be converted into value-added agro-textile mulch for improved crop production. The jute non-woven of different GSM and different blends with caddis can be manufactured through garneting cum cross lapping followed by needle punching [22].

Studies showed the beneficial role of pure jute non-woven mulch of different thicknesses in improving the yield of capsicum and pointed gourd [24], mosambi and turmeric [25], broccoli [26] and French bean [27] over no mulch condition. However, no studies showed the effect of non-woven mulch prepared through the blending of jute caddis with pure jute fibers on crop production, and hardly anybody attempted to study nutrient uptake, fruit quality, and water use efficiency of summer tomato under jute non-woven agrotextile along with jute -caddis blended agrotextile. Tomatoes are one of India's most economically important vegetable crops, ranking second globally in production and cultivated areas [28]. Summer tomatoes are rich in antioxidants, making them valuable for human consumption and health benefits [29], crucial in ensuring food security and addressing nutritional needs. A comprehensive understanding of the effect of physical and hydrological properties of non-woven jute on important hydrothermal and physical parameters of soil and overall crop growth is yet to be explored.

Therefore, the present investigation was undertaken to explore, in detail, a) to understand the effect of physical and hydrological properties of jute non-woven on important hydrothermal and physical parameters of soil and b) the efficacy of non-jute woven mulch along with jute caddis blended mulch on soil hydrothermal, microbiological properties, crop growth, nutrient uptake, water use efficiency, yield, and fruit quality of summer tomato crop over alluvial soil of Eastern India.

## 2 Materials and methods

### 2.1 Preparation of jute non-woven Mulch

Agrotextile mulch from jute non-woven was fabricated through the blending of 50 % pure jute and 50 % caddis (waste jute) on a weight basis having different GSM through garneting cum cross lapping followed by needle punching. Jute non-woven mulch of six different types with 100 % jute (100:0) of 250, 450, and 650 GSM, and the blend of pure jute with caddis (50:50) of 250, 450, and 650 GSM was developed at ICAR-NINFET, Kolkata, India for the field experiment (Table 1).

**Table 1:** Treatment details used in the experiment.

Treatment	Treatment Details
250, 100:0 (T1)	250 GSM, 100:0 (Pure Jute Fibre = 100 %: caddis = 0 %)
450, 100:0 (T2)	450 GSM, 100:0 (Pure Jute Fibre = 100 %: caddis = 0 %)
650, 100:0 (T3)	650 GSM, 100:0 (Pure Jute Fibre = 100 %: caddis = 0 %)
250, 50:50 (T4)	250 GSM, 50:50 (Pure Jute Fibre = 50 %: Caddis = 50 %)
450, 50:50 (T5)	450 GSM, 50:50 (Pure Jute Fibre = 50 %: Caddis = 50 %)
650, 50:50 (T6)	650 GSM, 50:50 (Pure Jute Fibre = 50 %: Caddis = 50 %)
Plastic (T7) (Positive Control)	Black Polyethylene Mulch, 50 μm [30]
Rice Straw (T8) (Positive Control)	Rice Straw applied @ 1 kg/m^2^ [31]
Control (T9) (Absolute Control)	No mulch

### 2.2 Field trials

**Fig. 1:**
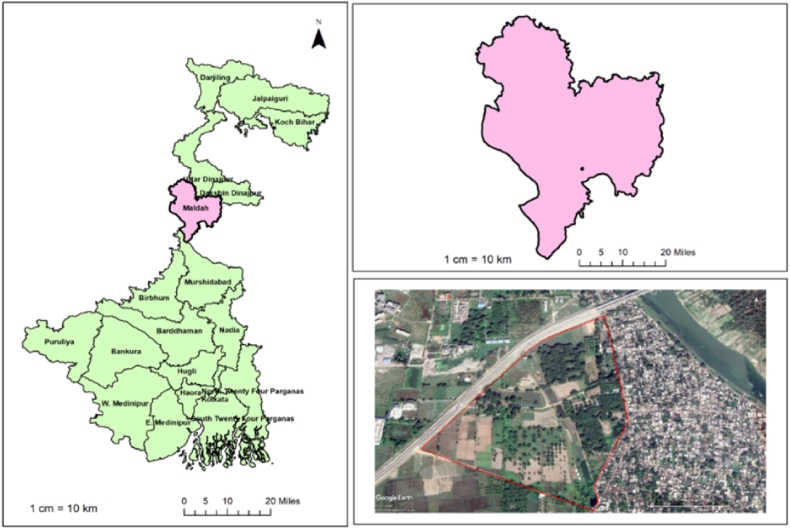
Geolocation of Experimental site at ICAR-CISH RRS, Malda, and West Bengal, India.

**Fig. 2:**
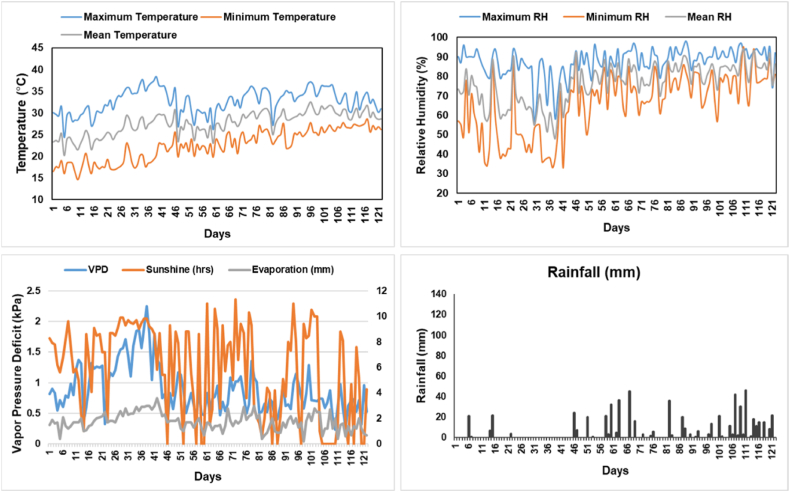
Weather Parameters during crop growing season.

Afield experiment was conducted in the summer months (March–June) of 2020 and 2021 at ICAR-Central Institute of Sub-tropical Horticulture (ICAR-CISH) Regional Station, Malda, West Bengal, India, which is located between 24.98° N and 88.15°E with an average altitude of 25m above mean sea level (Fig. 1). According to agro-climatic zonation by the Planning Commission of India, Malda district is a part of both the old alluvial zone (WB-3) and new alluvial zone (WB-4) with hot sub-humid to humid eco-sub region having tropical climate with annual rainfall around 1546 mm with average rainy days of 76 (SW monsoon: June to September, & NE monsoon: October to December) (West Bengal 10-Malda-31.12.2011 (agricoop.nic.in)). Rainfall amounts of 441.9 and 258.5 mm were received during the crop-growing season of 2020 and 2021, respectively. The weather data are presented in Fig. 2.

The experimental fields were characterized by deep, well-drained soil with a neutral pH 7.1 and an EC of 0.45 dS/m. The study followed a randomized block design with nine treatments (T1 to T9), each replicated in 5.0 m × 4.0 m plots three times. Jute non-woven geotextiles were placed on the beds of the experimental plots during planting according to the treatments, and tomato seedlings were transplanted by creating small holes in the mulching mat. For two seasons, nursery healthy seedlings with 3–4 mature leaves (4–5 weeks old) were transplanted in each experimental plot on March 3, 2020 and February 28, 2021. After transplanting, the crop cycle lasted 115 days (2020) and 120 days (2021).

### 2.3 Characterization of jute non-woven Mulch properties

#### 2.3.1 Chemical analysis of jute non-woven fabrics, rice straw

The chemical constituents of mulching materials, such as lignin, hemicellulose, fat, wax, and ash, were estimated using the TAPPI Method (1971) [32] and the α-cellulose content using the modified method of Sarkar et al. [33]. The pectin content was calculated using Dasgupta et al. [34]. The physicochemical properties of mulching materials are presented in Table 2.

**Table 2:** Physico-chemical properties of mulching materials.

Components	Non-woven Jute Agro-textile Mulch	Rice straw	Plastic
**α-Cellulose**	61.1 ± 2.76	34.79 ± 1.07	• Low-density polythene • Ethylene monomer was used to produce thermoplastic mulch, which exhibits a density falling within the range of 0.810–0.825 with a thickness of 50 μm
**Hemicellulose**	22.35 ± 0.93	26.13 ± 0.79	
**Lignin**	12.97 ± 0.47	10.66 ± 0.39	
**Fat and wax**	1.20 ± 0.024	5.15 ± 0.23	
**Nitrogenous matter**	1.65 ± 0.039	0.76 ± 0.031	
**Ash content**	0.73 ± 0.011	8.17 ± 0.35	

Data are Mean ± SEM.

#### 2.3.2 Tensile and hydro-physical properties of jute non-woven mulch

The tensile properties of the fabric were measured following ASTM D5035-23 standards [35] through an Instron Tensile testing machine (Model no. 5567). Ten measurements for each sample were taken to calculate tenacity, breaking elongation, and initial modulus [36]:

Tenacity (cN/tex) = Breaking load (cN)/Width of the test specimen (mm)∗Fabric mass per unit area (GSM).

#### 2.3.3 Hydro-physical properties of jute non-woven mulch

The Jute non-woven fabrics used as the mulching material were tested for their physical and mechanical properties for evaluation as mulching material. The area density of the fabrics was measured as per ASTM D5261 standard [37]. The thickness of the non-woven fabrics was measured with the ‘Prolific Thickness Tester’ with a top anvil diameter of 2.54 cm and with the application of 1.55 kPa pressure. The apparent opening size of the fabrics was measured as per ASTM D4751-23 [38]. Standard-sized glass granules were used for the purpose. The Thermal insulation value of the fabrics was calculated as per BS 4745 standard [39](Table 3).

**Table 3:** Physical, Thermal, and Hydraulic properties of Jute Non-woven fabrics.

Treatments	Thickness (mm)	Air Permeability (cm^3^/cm^2^∗s)	Thermal Insulation Value (TIV), Tog	Apparent Opening Size (AOS), O_95_ (mm)	Water flow rate (ml/s)	Transmissivity (m^2^/s)∗10-2
T1	2.48 ± 0.076	180.33 ± 8.07	1.55 ± 0.026	0.425 ± 0.007	6.07 ± 0.77	1.73 ± 0.032
T2	3.11 ± 0.093	100.67 ± 5.79	1.67 ± 0.017	0.250 ± 0.005	3.90 ± 0.58	1.54 ± 0.027
T3	4.10 ± 0.078	76.67 ± 3.29	1.91 ± 0.023	0.180 ± 0.002	3.58 ± 0.51	1.47 ± 0.023
T4	2.69 ± 0.167	126.87 ± 4.34	1.71 ± 0.046	0.425 ± 0.009	5.73 ± 0.71	2.13 ± 0.033
T5	4.30 ± 0.139	79.00 ± 5.34	2.19 ± 0.049	0.180 ± 0.003	5.11 ± 0.68	1.17 ± 0.019
T6	5.31 ± 0.163	56.80 ± 6.35	2.54 ± 0.31	0.150 ± 0.002	5.09 ± 0.65	1.08 ± 0.015

Data are Mean ± SEM.

#### 2.3.4 Biodegradability of jute non-woven fabrics

A degradation study of jute non-woven fabrics followed IS 1623: 1992 (reaffirmed in 2004) [40] and compared with non-degraded fabrics. FTIR spectra of fresh jute non-woven and degraded jute non-woven were measured using FT-IR Spectrometers (Bruker, USA).

### 2.4 Soil sampling& soil properties estimation

Soil samples were taken on three occasions: before planting, during the peak growth, and after the harvest. Around five soil cores were gathered randomly from every plot. The samples were split into two portions in the field and placed in plastic bags: one portion was preserved at 4 °C for the assessment of essential soil characteristics, while the other portion was air-dried in a well-ventilated area, ground and filtered through a <2-mm sieve to eliminate stones, root fragments, and organic matter before conducting chemical tests.

#### 2.4.1 Soil hydrothermal and physico-chemical properties

The soil moisture content of oven-dried (105 °C for 48 h) soil samples collected at 15, 30, 45, 60, 75, 90, and 105 DAT was determined gravimetrically [41] using the following formula:

Gravimetricwatercontent(%)=(Weightofwetsoil–Weightofdrysoil)/Weightofdrysoil∗100

Soil temperatures were measured at regular intervals coinciding with soil moisture measurement, from the beginning of the transplant of tomato seedlings until105 DAT using a digital thermometer with a probe of 115 mm long stainless steel at three depths (5, 15,and 30 cm)below the soil surface. Soil heat flux was measured using a heat flux sensor (Model-HFP01, Hukseflux Thermal Sensor BV, Netherlands), and soil unsaturated hydraulic conductivity was measured using a Minidisk Infiltrometer (Model-S, METER Group, USA).

The estimation methods of soil physiochemical parameters are given in Table 4.

**Table 4:** Soil parameters of the experimental field at planting.

Parameters	Methods/Process	Values/Remarks
Soil Type	USDA soil textural triangle [42]	Silty Loamy Soil (Alluvial Soil)
Clay (%)	The hydrometer method [43]	23.7 ± 0.88
Silt (%)	Same	41.1 ± 1.05
Sand (%)	Same	35.2 ± 0.97
pH (%)	Soil-to-water ratio of 1:2.5 [44]	6.83 ± 0.28
EC (μS/cm)	Same	35.03 ± 4.21
Available SOC (%)	Wet digestion method of Walkley and Black (1934) [45]	0.68 ± 0.04
Available N (kg/ha)	Alkaline potassium permanganate method [46]	101.54 ± 4.80
Available P (kg/ha)	Olsen method [47]	13.18 ± 0.83
Available K (kg/ha)	Neutral ammonium acetate method (soil/extractant 1:5) followed by Flame photometer estimation [44]	69.55 ± 2.74
Moisture (%)	Gravimetric method	19.40 ± 2.20
Soil Unsaturated Hydraulic conductivity (cm/s)	In-situ Tension Minidisk Infiltrometer method [48]	2.79 × 10^−04^
Bulk density (g/cm3)	Soil Core Method [49]	1.33 ± 0.011

Data are Mean ± SEM.

#### 2.4.2 Soil microbial population

The study aimed to investigate the influence of mulching on the population of bacteria, fungi, and actinomycetes at planting, 70 DAT (fruiting), and at the harvest (maturity). Soil samples from each treatment were collected and subjected to population enumeration using the serial dilution technique and pour plate method, as outlined by Aechra et al. [50] and Singh & Hamimed [51]. Nutrient agar, potato dextrose agar, and actinomycetes isolation agar media were used to quantify the bacteria, fungi, and actinomycetes population, respectively. Incubation of the plates was carried out at a temperature of 32 ± 2 °C, and the population of bacteria, fungi, and actinomycetes was assessed at 2, 5, and 7 days after inoculation, respectively. The results were expressed as colony-forming units (cfu) per gram of soil.

### 2.5 Plant growth measurement

Developmental evaluations were performed at 15, 30, 45, 60, 75, and 90 DAT, based on plant height and branch number, from 10 plants for each replication. The plants' height was measured with a 1-m ruler, considering the space between the soil surface and the apical meristem of the primary stem.

### 2.6 Nutrient uptake

At harvest time, samples of shoot and fruit tissues were obtained, dried at 70 °C, weighed, and crushed to 0.5 mm size. The samples were then analyzed for total nitrogen utilizing the micro-Kjeldahl digestion method [52]. Powdered leaf samples were digested in a 1:3 perchloric and nitric acid mixture to facilitate total phosphorus and potassium analysis. The determination of phosphorus (using vanadomolybdate) and potassium (via flame photometry) was carried out using Jackson's method [44]. The uptake of nitrogen, phosphorus, and potassium was calculated by multiplying the crop biomass (dry weight) by the concentrations of N, P, and K in the plant materials, resulting in the determination of uptake per hectare based on plant population.

### 2.7 Yield parameters and water use efficiency (WUE)

The computation of field water use efficiency involved assessing the biomass or yield of the crop (gm^−2^) relative to the quantity of water (cm) utilized. Weekly harvests were performed, and the resulting fruit yield was quantified as kg/m^2^.

### 2.8 Fruit quality analysis

The Brix meter assessed the concentration of soluble solids through refractometric readings (at 20 °C) of samples consisting of the pulps of twenty fruits per plot, harvested when fully ripe and then crushed. To determine the total phenolic content (TPC), the Folin-Ciocalteu reagent method, a reliable technique introduced by Singleton and Rossi [53] was employed and was quantified against a gallic acid standard curve (R^2^ = 0.99). Furthermore, the total flavonoid content was evaluated using the aluminium chloride (AlCl3) method developed by Zhishen et al. [54] and was calculated based on a quercetin standard curve (R^2^ = 0.99).

### 2.9 Financial ratio

Calculating a tomato production system's benefit-cost ratio (BCR) involves comparing the total net income generated to the total costs incurred. The BCR is a crucial indicator of the profitability of an investment with the following formula [55]:

BCR=TotalCosts/TotalNetIncome

If the BCR is greater than 1, the investment is profitable. If it's less than 1, it's not profitable.

### 2.10 Statistical analysis

Data is presented in tables as arithmetic mean ± standard error of the mean. Statistical significance was tested using the R program (version 3.6.1) through one-way ANOVA for field experimental data. Duncan's Multiple Range Test (DMRT) was used for treatment difference and was considered statistically significant when P ≤ 0.05 [56]. Two-way ANOVA followed by Tukey HSD was applied for parameters under two factors and presented in bar graphs. Correlation analyses were carried out using R software.

## 3 Results and discussion

### 3.1 Hydro-physical properties of jute non-woven fabric

The results on different mulch properties showed that the tenacity of the non-woven materials with the caddis was almost similar or sometimes better due to the backing cloth, which resulted in the lower breaking elongation values of the non-woven made with caddis. Thickness is higher in 50:50 (Jute: Caddis) non-woven mulches than in 100:0 (Jute: Caddis) non-woven, resulting in more thermal insulation and less air permeability. There were no significant differences in apparent opening size for both types of non-woven fabrics. The areal density, thickness, air permeability, and thermal insulation values are given in Table 3. It can be observed that the non-woven fabric made from fresh jute fibre is more compact and has lower thickness compared to the fabric made from jute caddis. As the caddis is short fibres, they are difficult to compact by needle punching and this phenomenon produces more hollowness in the fabric structure. This phenomenon is also observed in the thermal insulation values. The short caddis also tries to fill the gaps inside the structure, especially the needled area. Thus, the air permeability and the apparent opening size (AOS) of the non-woven fabrics with jute caddis are lower than those produced from fresh jute fibre.

### 3.2 Biodegradability

#### 3.2.1 Tensile properties of the jute non-woven

The non-woven fabrics produced from blended jute caddis required a very open structure backing cloth for production, which becomes a part of the non-woven fabric. The tensile properties of the jute non-woven from caddis depend on the backing cloth, as observed in the table. As expected for the jute fabric produced from fresh fiber, the strength of the fabric increases with an increase in the areal density. The mulch material of 250, 50:50 (T4) showed the lowest strength. The tensile properties of the jute non-woven fabrics are given in Table 5.

**Table 5:** Tensile properties of fresh and degraded Jute Non-woven fabrics.

Treatments	Fresh Jute Non-woven			Degraded Jute Non-woven		
	Tenacity (cN/tex)	Breaking elongation (%)	Initial Modulus (N/mm^2^)	Tenacity (cN/tex)	Breaking elongation (%)	Initial Modulus (N/mm^2^)
T1	0.286 ± 0.047	35.514 ± 2.948	5.384 ± 0.446	0.16 ± 0.027	15.47 ± 2.13	3.50 ± 0.500
T2	0.544 ± 0.062	29.124 ± 1.966	12.266 ± 1.520	0.23 ± 0.025	8.20 ± 1.59	5.01 ± 1.117
T3	0.792 ± 0.063	25.408 ± 0.893	19.472 ± 1.990	0.29 ± 0.024	4.15 ± 0.29	7.47 ± 1.312
T4	0.172 ± 0.014	33.816 ± 2.694	1.388 ± 0.122	0.08 ± 0.017	9.18 ± 1.37	0.80 ± 0.140
T5	0.278 ± 0.015	20.26 ± 2.266	3.576 ± 0.430	0.11 ± 0.016	6.26 ± 1.21	2.41 ± 0.686
T6	0.354 ± 0.024	16.168 ± 1.922	5.634 ± 1.012	0.16 ± 0.030	3.00 ± 0.58	2.71 ± 0.678

Data are Mean ± SEM.

#### 3.2.2 FTIR spectra analysis

**Fig. 3:**
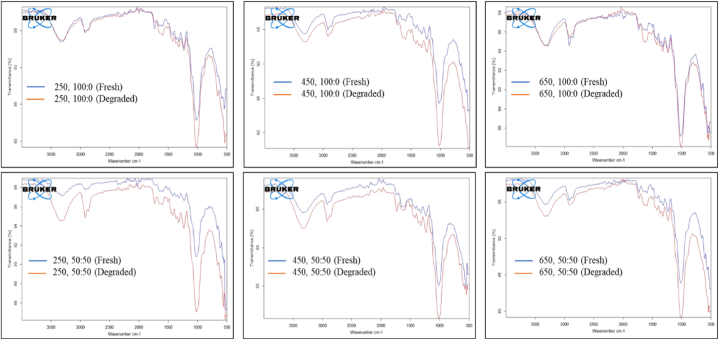
FTIR spectra of fresh and degraded jute non-woven mulch treatments.

The absorption peaks in the FTIR spectra of jute non-woven fabric can provide insights into its biodegradability. The FTIR spectral analysis conducted on degraded non-woven jute agro-textile mulch of different GSM and blends revealed significant variations in peak intensities, shifts of peak positions, and the appearance/disappearance of specific peaks corresponding to cellulose (C-O-C group), lignin (Ar-OH group), hemicelluloses (C=O group), and other components (O-H group and C=C group), as presented in Fig. 3 and Table 6. These changes are related to modifications in the chemical composition of the functional groups of the tested samples due to the combined effects of sunlight, moisture, and heat [26]. It was observed that the rate of degradation of non-woven jute mulch varied based on its thickness and composition. Agro-textile mulch of 650 GSM, made up of 100 % jute, was resistant to degradation, whereas 250 GSM, made up of jute and caddis, was more susceptible to degradation. The nature of changes in the peak intensity of the C-O-C stretching vibration band at 1010–1020 cm⁻^1^ and the O-H stretching band at 3298–3340 cm⁻^1^ (Table 6) showed indicative progressive degradation of the cellulose component, possibly occurring due to hydrolysis and enzymatic activities [57]. Lignin, providing structural support, exhibited alterations in the Ar-OH stretching vibration at 2898–2937 cm⁻^1^ (Table 6; Fig. 3), suggesting potential degradation of lignin-rich regions. In contributing to structural integrity, Hemicellulose displayed changes in the C=O stretching vibration, indicating degradation-related alterations. Due to oxidation, carbonyl groups showed changes in intensity through the C=O stretching vibration at 1660–1732 cm⁻^1^ [58]. Hydroxyl groups, abundant in cellulose, lignin, and hemicellulose, displayed shifts or changes in intensity in both O-H stretching at 3340 to 3262 cm⁻^1^ and bending vibrations at 1217–1232 cm⁻^1^ (Table 6), reducing their peak intensity due to hydrolytic degradation processes [59]. Alterations in the intensity of peaks related to C=C stretching vibrations suggested changes in the aromatic structure due to degradation processes at 1580–1725 cm⁻^1^ (Table 6), providing the degradation pattern of non-woven jute agro-textile essential for assessing durability study for repeated application as a mulch.

**Table 6:** FTIR absorption bands of fresh and degraded Jute Non-woven fabrics.

Treatments	Fresh Jute Non-woven					Degraded Jute Non-woven				
	Absorption Band (cm-1)					Absorption Band (cm-1)				
	Z1	Z2	Z3	Z4	Z5	Z1	Z2	Z3	Z4	Z5
250, 100:0 (T1)	3343	2898	1580	1226	1017	3334	2900	1588	1231	1013
450, 100:0 (T2)	3312	2904	1582	1221	1006	3262	2913	1590	1232	1012
650, 100:0 (T3)	3284	2912	1587	1226	1014	3269	2937	1632	1229	1009
250, 50:50 (T4)	3306	2900	1721	1217	1012	3288	2917	1725	1224	1007
450, 50:50 (T5)	3334	2908	1721	1224	1014	3330	2911	1723	1226	1009
650, 50:50 (T6)	3330	2912	1723	1223	1008	3327	2910	1725	1225	1005

### 3.3 Effect of Mulch treatments on soil hydrothermal properties

#### 3.3.1 Soil temperature and soil heat flux

**Fig. 4:**
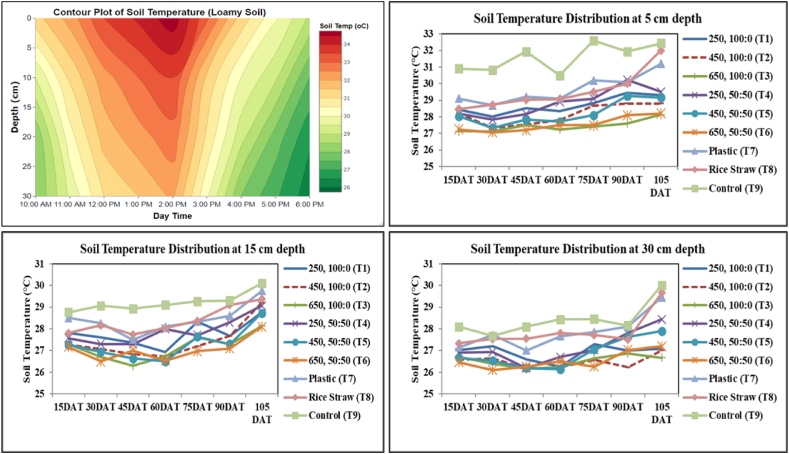
Diurnal soil temperatures and temporal variation of soil temperature under different mulch treatment.

**Fig. 5:**
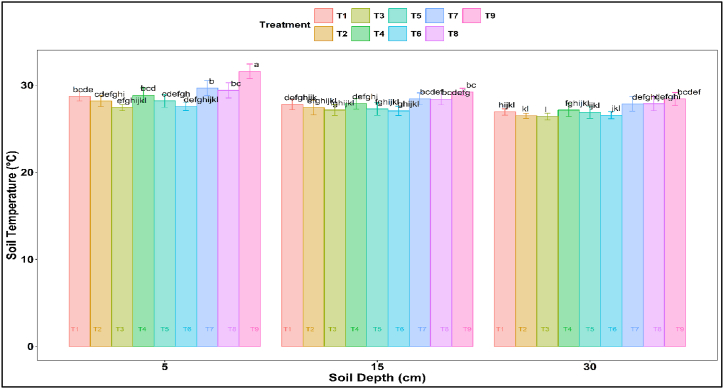
Variation of temperatures at 5, 15, and 30 cm soil depth under different mulch treatment.

Diurnal variation of soil temperature showed that it reaches a maximum around 1–2 PM and decreases as the depth increases (Fig. 4). Bimonthly soil temperature data exhibit that the soil temperature of the mulched plot was lower over the control for all three depths (5, 15, and 30 cm), and it was the lowest for 650, 100:0 & 50:50 (T3& T6)(Fig. 5). The temporal data recorded from the experimental field exhibits that the ranges of reduction in soil temperature at 5, 15, and 30 cm soil depth are 6–13 %, 10–14 %, and 12–16 %, respectively. The maximum reduction (17 %) in soil temperature was observed in T3 at 30 cm soil depth on 45 DAT, and the minimum reduction (2 %) in soil temperature was observed in T8 at 30 cm soil depth on 75 DAT over T9 (control) (Fig. 4). The result showed that soil temperature under plastic mulch treatment was considerably higher than that of jute non-woven of different types for all soil depths and dates. Xu et al. [60] also found that organic mulches reduced soil temperature and maintained higher soil moisture levels than black plastic mulch. Noor et al. [61] reported decreased soil temperature under straw mulch treatment. Amare and Desta [3] highlighted that colored plastic mulches, mainly black and blue, can increase soil temperature in the summer.

In the Indian subcontinent, where summer temperatures can be intense, using plastic mulch, mainly black plastic mulch, may lead to a more significant increase in soil temperature than organic mulches. Our study also showed higher soil temperature under plastic over straw mulch and jute non-woven mulch of different types for topsoil and sub-soils. The decrease in soil temperature for T3 and T6 is statistically significant over plastic, rice straw, and control (Fig. 5). This indicates that jute non-woven mulches may provide a more favorable environment (1.5–2.0 °C lower than plastic mulch) for crops sensitive to high temperatures during the summer. Jute non-woven with caddis exhibits performance for soil temperature similar to non-woven mulch with fresh jute.

#### 3.3.2 Soil moisture and soil unsaturated hydraulic conductivity

**Fig. 6:**
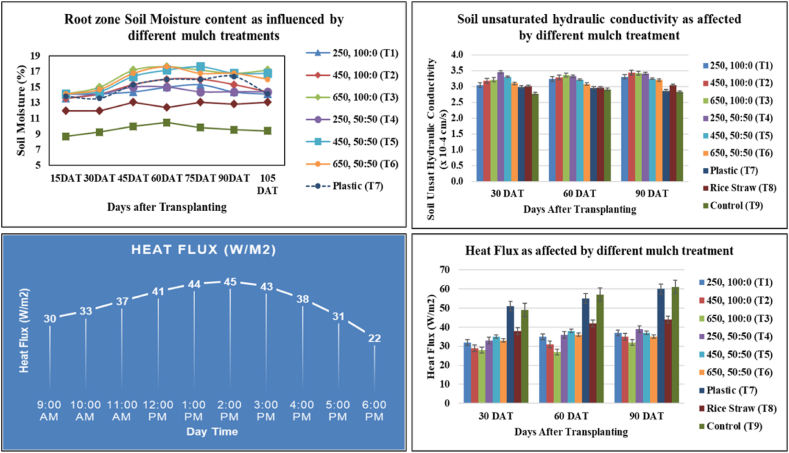
Root zone soil moisture, soil unsaturated hydraulic conductivity, and soil heat flux under different mulch treatment.

The result indicated that root zone soil moisture was lowest in no mulch compared to mulched treatments (30–71 % higher) in all the dates (Fig. 6). In various studies [[62], [63], [64]], researchers have observed elevated surface soil moisture levels and lower evaporation rates due to enhanced soil moisture retention under mulch involving cooler soil temperatures and decreased transpiration from lower weed population [61,65]. Results showed that maximum soil moisture content was observed on 75 DAT at the peak growth stage of tomato crops for all treatments (Fig. 6). T3 & T5 showed increased soil moisture content of68 & 71 % over control, 7.5 & 9.3 % over plastic. However, other jute non-woven mulch (T1, T2 & T4) have lower moisture (0.4–4.0 %) than plastic (Table 6). Jute non-woven with caddis exhibits performance for soil moisture similar to non-woven mulch with fresh jute.

Thermal properties are fundamental to determining heat transfer through fabrics [66]. Soil heat flux and soil temperature showed a similar diurnal variation trend, reaching a maximum of around 1–2 PM. Soil heat flux was maximum in plastic mulch, followed by a control plot, and all jute non-woven mulch showed lower heat flux values by a reduction of 33–47 % over plastic and 34–48 % over no mulch. Soil unsaturated hydraulic conductivity was higher in the mulched plot than in the control (Table 7).

**Table 7:** Soil hydrothermal characteristics as affected by different mulch treatments for whole crop duration.

Treatments	Soil moisture (%)	Soil Heat Flux (W/m2)	Soil Unsaturated Hydraulic conductivity (x 10^−4^ cm/s)
250, 100:0 (T1)	14.42 ± 0.22 ^b^	34.67 ± 1.45 ^cd^	3.20 ± 0.08 ^bc^
450, 100:0 (T2)	14.97 ± 0.36 ^b^	31.67 ± 1.76 ^de^	3.30 ± 0.08 ^ab^
650, 100:0 (T3)	16.43 ± 0.50^a^	29.00 ± 1.53^e^	3.34 ± 0.06 ^ab^
250, 50:50 (T4)	14.49 ± 0.14 ^b^	36.00 ± 1.73^c^	3.27 ± 0.04^a^
450, 50:50 (T5)	16.17 ± 0.52^a^	36.67 ± 0.88^c^	3.26 ± 0.03 ^abc^
650, 50:50 (T6)	16.11 ± 0.48^a^	34.67 ± 0.88 ^cd^	3.23 ± 0.04 ^cd^
Plastic (T7)	15.04 ± 0.44 ^b^	55.33 ± 2.60^a^	2.94 ± 0.04 ^ef^
Rice Straw (T8)	12.64 ± 0.19^c^	41.33 ± 1.76 ^b^	3.01 ± 0.02 ^de^
Control (T9)	9.61 ± 0.22^d^	55.67 ± 3.53^a^	2.84 ± 0.04^f^

(Data are means ± SEM with three replications, and the letters a, b, c, d, e, and f indicate Duncan's grouping of treatment differences. Means with the same letter are not significantly different.).

### 3.4 Effect of Mulch treatments on microbial population

**Fig. 7:**
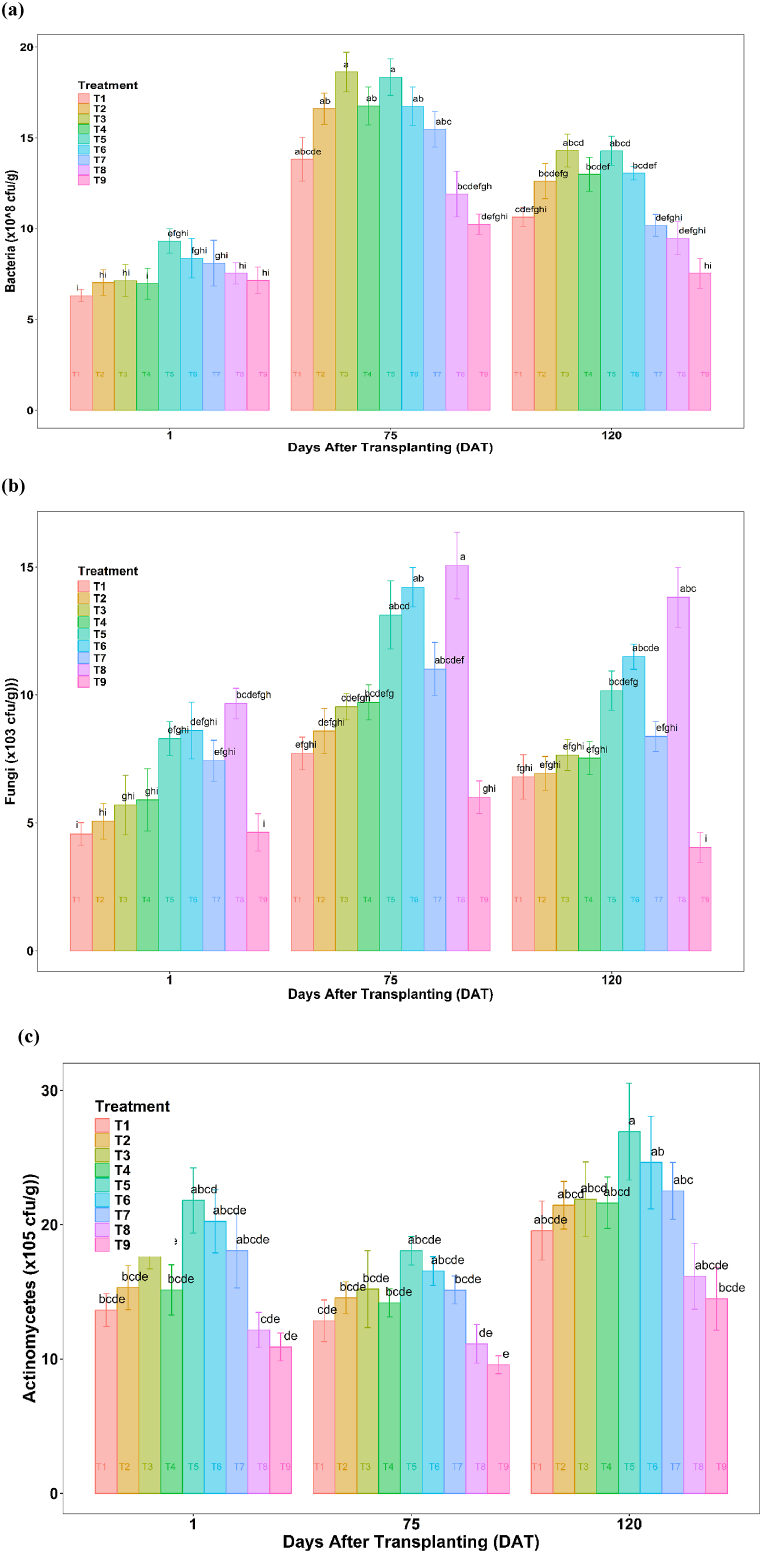
Microbial population in soils during planting, Fruiting (75 DAT), and Harvesting (120 DAT) of summer tomatoes as affected by different mulch treatments.

The experimental data showed that the initial bacterial population was lower at the planting, which increased to the maximum during the peak crop growth stage (75 DAT) and decreased at the harvesting stage (Fig. 7). The bacterial populations increased significantly in the soil from planting to 75 DAT in all the treatments due to favorable temperature, humidity, and anaerobic conditions under jute non-woven mulches which is in line with the observation by Subba [67]. Data recorded on essential growth stages of tomato crop at planting, 75 DAT, and harvesting revealed that a maximum increase (160 %) from planting to 75 DAT in bacterial population was observed in T3 (650 GSM, 100:0) followed by 140 % increase in T4 (250 GSM, 50:50) and 136 % increase in T2 (450 GSM, 100:0). Throughout planting to harvesting, there was a declining trend of soil bacterial population due to occurrence of high soil temperature and lower soil moisture content. Studies have also indicated that mulching can promote bacterial colonization and increase carbon retention in the form of bacterial necromass, potentially affecting the bacterial populations in the soil [68,69]. The inhibitory effect of root exudates on bacteria by grasses was also reported by Gopalakrishnan et al. [70] and the lower bacterial population was found during summer [71]. However, the actinomycetes and fungal population in soil increased from planting to harvesting. The lowest count of the actinomycetes population was recorded in T8 (rice straw), but the fungi population increased significantly in rice straw. At 75 DAT, it was observed that the population of actinomycetes under almost all the treatments decreased from its initial value and, after that, increased gradually during the harvesting period, which may be due to increased carbon availability at the maturity stage [72]. Under all the treatments, the fungal population in the soil increased initially and gradually decreased toward the harvesting stage. There was an overall increase of microbial population by 35–117 % for jute non-woven over no mulch and 6–70 % over plastic mulch.

### 3.5 Effect of Mulch treatments on weed suppression

**Fig. 8:**
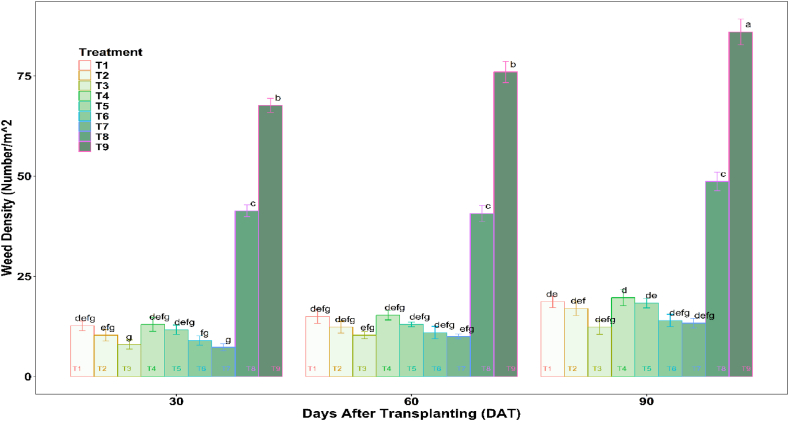
Weed density as influenced by Mulch Treatments.

The experiment data showed that weed density, i.e., number of weeds per square meter, was significantly lower in mulched treatments over control (T9). Data recorded on critical growth stages at 30, 60, and 90 DAT revealed that the weed population was lowest in polythene mulch, T7 at 30 and 60 DAT, and T2 at 90 DAT (Fig. 8). The reduction in weed density was in the range of 39–89 %, 40–85 %, and 28–82 % at 30, 60, and 90 DAT, respectively. The reduction in jute non-woven mulch at the early stages of crop growth was more significant than in the later stages. This may be because the biodegradable jute non-woven mulch starts degrading at later crop growth stages. The use of mulches as a physical barrier effectively suppressed the emergence of weeds, although this effect was transient and diminished as the mulches underwent decomposition. Studies by Wilen et al. [73] and Subba [67] also reported similar observations.

### 3.6 Crop growth parameters under different Mulch treatments

**Fig. 9:**
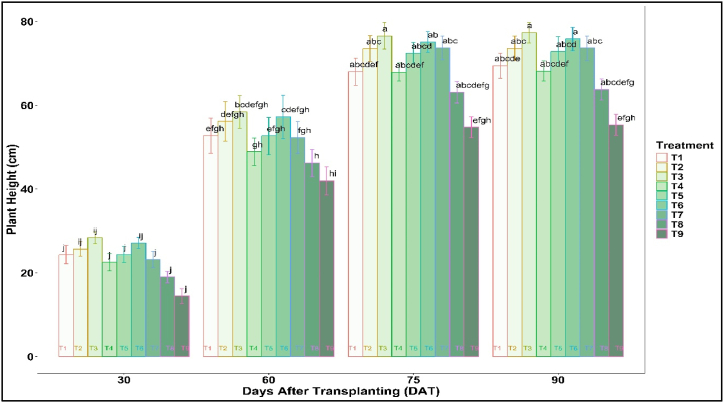
Plant height of summer tomato as influenced by mulch treatment.

The experimental data showed that plant height was significantly higher in mulched treatments over no mulch (T9) (Fig. 9). Data recorded on critical growth stages at 30, 60, 75, and 90 DAT revealed that plant heights peak at 90 DAT and become almost stagnant. Plant height was highest in T3 (77.33 cm), followed by T6 (75.87), and plastic (73.67). The smallest height was seen in no mulch (T9). The increase in plant height for jute non-woven over no mulch was around 23–39 % and 3–5% (for T3 and T6) over plastic mulch.

### 3.7 Nutrient uptake

Mulching has been shown to significantly impact summer tomatoes' nutrient uptake and yield. The result showed that the uptake of essential nutrients (N, P, K) is considerably higher in mulch treatment over no mulch (Table 8). Nitrogen uptake in jute non-woven mulch was higher over plastic mulch by (0.5–15) % and over no mulch by 19–36 %. Similarly, an increase in P and K uptake over no mulch was recorded as 47–64 % and 25–30 %, respectively, and over plastic mulch as 6–18 % and 3–7% respectively. The nutrient uptake was maximum in T3 followed by T2 and T6. Shehata et al. [74] also supported this finding by highlighting that mulching can reduce nutrient loss by leaching, retaining soil heat and moisture, and increasing the sink capacity of tomato fruits, ultimately enhancing nutrient uptake and early yield. Peng-fei et al. [65] found that mulching can reduce nutrient leaching by slowing irrigation infiltration, thereby maintaining soil nutrients for tomato growth. Results indicate that mulch treatments play a crucial role in improving the nutrient uptake of summer tomatoes by enhancing soil conditions, reducing nutrient loss, and promoting better vegetative growth.

**Table 8:** Nutrient uptake as influenced by different mulch treatments.

Treatments	Nutrient Uptake (kg/ha)		
	N	P	K
**250, 100:0 (T1)**	100.80 ± 4.46 ^ab^	13.61 ± 0.61 ^ab^	72.80 ± 3.05^a^
**450, 100:0 (T2)**	108.46 ± 3.72^a^	14.86 ± 1.27^a^	72.87 ± 1.91^a^
**650, 100:0 (T3)**	115.10 ± 5.31^a^	15.17 ± 1.15^a^	74.55 ± 2.19^a^
**250, 50:50 (T4)**	103.12 ± 3.51 ^ab^	13.78 ± 0.78 ^ab^	72.15 ± 2.59^a^
**450, 50:50 (T5)**	104.95 ± 4.96 ^ab^	13.87 ± 0.87 ^ab^	72.52 ± 2.83^a^
**650, 50:50 (T6)**	107.05 ± 5.25 ^ab^	13.98 ± 0.78 ^ab^	72.12 ± 2.54^a^
**Plastic (T7)**	100.23 ± 5.41 ^ab^	12.87 ± 0.64 ^ab^	69.72 ± 2.07 ^ab^
**Rice Straw (T8)**	91.56 ± 5.92 ^bc^	11.48 ± 0.57 ^bc^	61.70 ± 3.55 ^bc^
**Control (T9)**	84.57 ± 4.67^c^	9.23 ± 0.87^c^	57.47 ± 3.97^c^

(The Data are means ± SEM with three replications, and the letters a, b, c, d, and e indicate the Duncan grouping of treatment differences. Means with the same letter are not significantly different.).

### 3.8 Correlation analysis

**Fig. 10:**
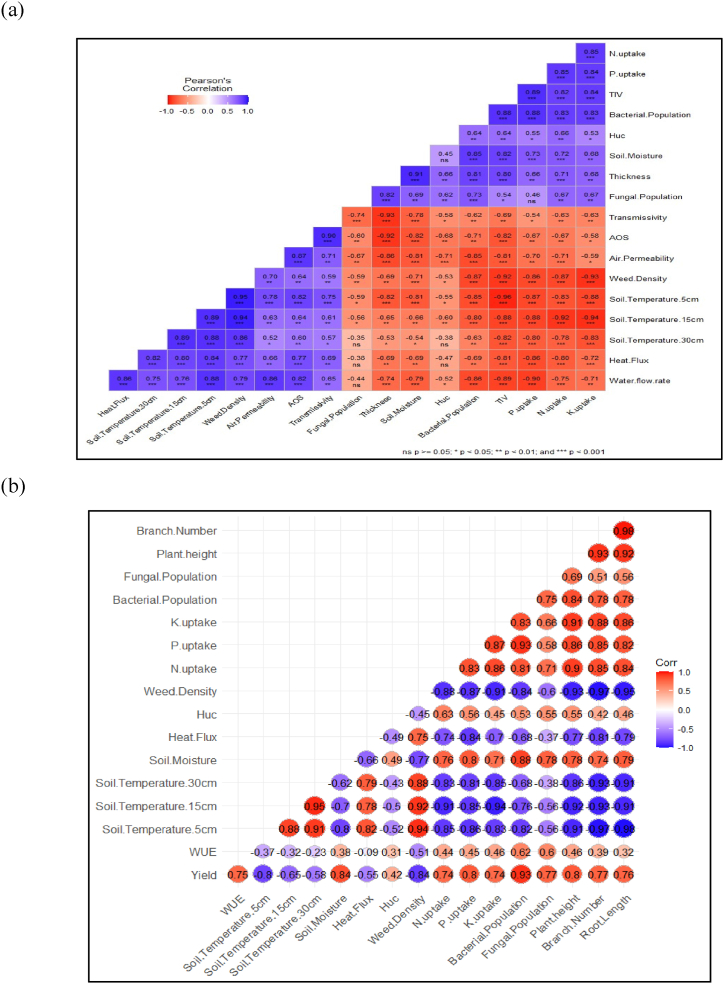
(a) Correlogram showing basic hydro-physical properties of jute non-woven fabrics with soil, microbial properties, weed density, and nutrient uptake, (b) Correlogram showing correlation between soil, microbial properties, weed density, and nutrient uptake with crop yield and WUE.

The correlation analysis showed that soil moisture content increases with higher thickness (r = 0.91) and higher TIV (r = 0.82) of jute non-woven fabrics. In contrast, it decreases with higher transmissivity (r = 0.75), AOS (r = 0.82), Air permeability (r = 0.81), and water flow rate(r = 0.79). Increased soil moisture content enhances N-uptake (r = 0.72), P-uptake (r = 0.73), K-uptake (r = 0.66), bacterial population (r = 0.85), and fungal population (r = 0.69). Soil temperatures at 5, 15,and 30 cm and heat flux decreased with increasing soil moisture content (Fig. 10a). Tomato yield and crop growth parameters are positively correlated with soil moisture content (r = 0.84), nutrient uptake (r = ∼0.8), and microbial population (r = 077-0.93). WUE was positively correlated with soil moisture (r = ∼0.4), microbial population (r = ∼0.6), nutrient uptake (r = ∼0.45), crop growth parameters (r = 0.4–0.46), and unsaturated hydraulic conductivity (r = 0.31), and negatively correlated with soil temperatures (r = 0.23–0.37), and weed density (r = 0.51) (Fig. 10b).

### 3.9 Yield parameters

The experiment revealed highly significant variation in fruit parameters and tomato yield under different mulching treatments (Table 9). Increase in fruit weight in mulched treatments was in the range of 45–106 % over no mulch (T9), with maximum improvement (106 %) observed in jute non-woven (T3) followed by 101 % increase in (T2) and 98 % increase in (T4). The Increase in fruit height in mulched treatments was in the range of 16–29 % over control (T9) with maximum improvement (29 %) observed in T3 and T2 followed by a 28 % increase in T6. Similarly, the increase in fruit diameter for jute non-woven over plastic mulch was 3–11 %. Overall improvement in fruit diameter in mulched treatments was in the range of 21–37 % over no mulch (T9), with maximum improvement (∼37 %) observed in T3, and in T2 and a 29 % increase in T6. The increase in yield for jute non-woven over no mulch and plastic was 34–84 %, and 4–28 %, respectively. The increase in water use efficiency (WUE) was highest in T3 (44 % over control and 21 % over plastic), followed by T6 (37 % over no mulch and 15 % over plastic), and T5 (34 % over no mulch and 12 % over plastic). The overall increase in WUE for mulched treatments was 14–44 % over no mulch (T9). The improvement in jute non-woven mulch treatments, especially T3 over rice straw, polythene mulch, and no mulch in the experiment, attributed to the beneficial effect of increased moisture reserve, improved soil temperature modulation, increased microbial population, higher nutrient uptake along with the highest reduction in weed density.

**Table 9:** Fruit characters, Yield parameters, and Water Use Efficiency as influenced by different mulch treatments.

Treatments	Fruit Weight (g)	Fruit Length (cm)	Fruit Diameter (cm)	Yield (kg/m2)	WUE (kg/m3)
**250, 100:0 (T1)**	105.92 ± 1.77 ^**bc**^	4.84 ± 0.04 ^**abcd**^	6.08 ± 0.17 ^**ab**^	14.33 ± 0.88 ^**c**^	10.1 ± 0.84 ^**cd**^
**450, 100:0 (T2)**	116.91 ± 3.26 ^**ab**^	5.07 ± 0.10 ^**ab**^	6.44 ± 0.35 ^**a**^	16.30 ± 1.15 ^**bc**^	10.77 ± 0.15 ^**bc**^
**650, 100:0 (T3)**	120.20 ± 2.15 ^**a**^	5.09 ± 0.03 ^**a**^	6.46 ± 0.29 ^**ab**^	19.67 ± 0.87 ^**a**^	11.93 ± 0.15 ^**a**^
**250, 50:50 (T4)**	114.77 ± 2.57 ^**ab**^	5.06 ± 0.06 ^**abc**^	6.11 ± 0.15 ^**ab**^	16.03 ± 0.67 ^**b**^	11.03 ± 0.25 ^**ab**^
**450, 50:50 (T5)**	113.79 ± 3.72 ^**ab**^	4.83 ± 0.06 ^**bcd**^	6.01 ± 0.13 ^**ab**^	16.00 ± 0.98 ^**bc**^	11.10 ± 0.35 ^**ab**^
**650, 50:50 (T6)**	116.01 ± 3.24 ^**ab**^	4.94 ± 0.03 ^**de**^	6.20 ± 0.06 ^**ab**^	16.67 ± 0.81 ^**bc**^	11.33 ± 0.21 ^**bc**^
**Plastic (T7)**	99.49 ± 5.31 ^**c**^	4.81 ± 0.07 ^**cd**^	5.81 ± 0.21 ^**ab**^	15.33 ± 0.89 ^**bc**^	9.87 ± 0.60 ^**cd**^
**Rice Straw (T8)**	84.04 ± 3.81 ^**d**^	4.56 ± 0.14 ^**e**^	5.71 ± 0.23 ^**b**^	14.67 ± 0.33 ^**c**^	9.33 ± 0.42 d
**Control (T9)**	58.10 ± 2.25 ^**e**^	3.94 ± 0.15 ^**f**^	4.72 ± 0.26 ^**c**^	10.67 ± 0.69 ^**d**^	8.30 ± 0.20 ^**e**^

(Data are means ± SEM with three replications, and the letters a, b, c, d, e, and f indicate Duncan's grouping of treatment differences. Means with the same letter are not significantly different.).

### 3.10 Fruit quality parameters

Data showed improvement in fruit quality parameters like TSS, phenol, flavonoids, and lycopene content in jute non-woven mulched treatments over other treatments (Table 10). According to TSS utterance, the Brix is connected to the proportion of sugars (mainly glucose and fructose), which helps calculate sensory attributes such as taste, sweetness, and acidity. TSS content is higher in T4 (4.23 %) than in other mulching treatments. T8 (3.07 %) shows the lowest TSS content. The different mulching treatments of tomatoes contained 1.21–1.92 mg GAE/100g, and total phenolics content was observed in the order of T2>T3>T4>T5>T7>T1>T6>T8>T9. Among different mulching treatments of tomatoes, the lowest total phenolic content was found in the control (1.21 ± 0.35 mg GAE/100g), followed by T8. The total phenolic content of tomatoes in T2 was high compared to that of the other mulching treatment (Table 10). Among different bioactive compounds, lycopene is the primary carotenoid found in tomatoes. Studies have shown that the cultivation environment, such as high tunnel covering and soil mulching, can impact the lycopene content in tomatoes [75]. In our study, lycopene content ranged from 4.11 to 5.93 mg/100g. The lowest lycopene content was observed in no mulch (T9) among different mulching treatments, whereas T2 showed the highest (Table 10). Overall, T2, T3, and T4 showed better nutritional values than other treatments.

**Table 10:** Fruit quality parameters of summer tomatoes under different mulch treatments.

Treatments	TSS (%)	Total phenol (mg GAE/100g)	Total Flavonoids (mg QE/100g)	Lycopene content (mg/100g)
**250, 100:0 (T1)**	3.33 ± 0.38 ^bc^	1.51 ± 0.21 ^**ab**^	6.50 ± 0.84 ^abc^	4.91 ± 0.31 ^**ab**^
**450, 100:0 (T2)**	3.77 ± 0.41 ^abc^	1.92 ± 0.42 ^**a**^	7.95 ± 0.91^a^	5.93 ± 0.49 ^**a**^
**650, 100:0 (T3)**	3.70 ± 0.12 ^abc^	1.74 ± 0.38 ^**ab**^	7.55 ± 0.92 ^ab^	5.79 ± 0.47 ^**ab**^
**250, 50:50 (T4)**	4.23 ± 0.29^a^	1.68 ± 0.33 ^**ab**^	7.73 ± 0.95 ^ab^	5.67 ± 0.53 ^**ab**^
**450, 50:50 (T5)**	3.70 ± 0.20 ^abc^	1.62 ± 0.39 ^**ab**^	6.05 ± 0.55 ^abc^	4.85 ± 0.57 ^**bc**^
**650, 50:50 (T6)**	3.50 ± 0.12 ^abc^	1.37 ± 0.23 ^**ab**^	6.29 ± 0.59 ^abc^	4.77 ± 0.63 ^**bc**^
**Plastic (T7)**	4.00 ± 0.06 ^ab^	1.52 ± 0.32 ^**ab**^	6.19 ± 0.96 ^abc^	4.56 ± 0.42 ^**bc**^
**Rice Straw (T8)**	3.07 ± 0.13^c^	1.31 ± 0.34 ^**ab**^	5.79 ± 0.47 ^bc^	4.31 ± 0.44 ^**bc**^
**Control (T9)**	3.27 ± 0.12 ^bc^	1.21 ± 0.35 ^**b**^	5.73 ± 0.54 ^bc^	4.11 ± 0.55 ^**bc**^

**(**The Data are means ± SEM with three replications, and the letters a, b, and c indicate Duncan's grouping of treatment differences. Means with the same letter are not significantly different.).

## 4 Economic viability

Jute non-woven fabric with 100 % pure jute of 250, 450, and 650gsm costs around $0.35/-, $ 0.47/-, and $ 0.64/- per square meter in the Indian market. Whereas jute non-woven fabric with a blend of 50 % pure jute and 50 % caddis of 250, 450, and 650 gsm costs around $ 0.30/-, $ 0.44/-, and $ 0.60/-, per square meter. The study showed a net extra profit of $ 6590/- per ha of summer tomato for Jute non-woven mulch (T3) over no-mulch and $ 840/- over plastic mulch, assuming a tomato price of $0.3/- per kg during summer. The benefit-cost ratio as analyzed is given in Table 11.

**Table 11:** Benefit -Cost Ratio of tomato production under different mulch treatments.

Sl. No.	Treatments	BC ratio
1.	250, 100:0 (T1)	1.79
2.	450, 100:0 (T2)	1.94
3.	650, 100:0 (T3)	2.28
4.	250, 50:50 (T4)	1.94
5.	450, 50:50 (T5)	1.92
6.	650, 50:50 (T6)	1.93
7.	Plastic (T7)	1.92
8.	Rice Straw (T8)	1.60
9.	Control (T9)	1.41

## 5 Conclusions

Various mulch material impacts tomato yield and fruit quality parameters due to differences in mulch properties and their interactions with soil, crop, and weather parameters. Among all the mulch materials, Jute non-woven 650 GSM, 100:0, followed by 250 GSM, 50:50, and 450 GSM, 100:0, showed significant differences over no mulch, straw, and plastic mulch, regarding tomato yield, fruit quality, crop growth, nutrient uptake, soil moisture, and microbial parameters. Every mulch treatment improved tomato yield, WUE, and fruit quality parameters over control. Total phenol, flavonoids, lycopene, and antioxidant parameters showed improved results under jute non-woven plot over control and plastic mulch. The better performance of jute non-woven mulch treatments, especially 650 GSM, 100:0 (T3) over rice straw, polythene mulch, and no mulch in the experiment, attributed to the fact that T3 treatment with lower air permeability, AOS, transmissivity, water flow rate, and higher thickness resulted in increased soil moisture content which in turn increases higher unsaturated hydraulic conductivity, improved nutrient uptake and microbial population along with improved soil temperature modulation and reduction in weed density. Jute non-woven mulch provides the most favorable soil condition for tomatoes and results in higher growth and yield of the crop. So, the study showed that this particular eco-friendly production system with jute non-woven mulch considerably improved tomato yield and fruit quality with improved nutritional values over traditional plastic mulch and no mulch production system and can be considered a potential, sustainable, and economical alternative to the ever-growing plastic perils in a crop production environment.

## CRediT authorship contribution statement

**Nilimesh Mridha:** Investigation, Funding acquisition, Formal analysis, Data curation, Conceptualization. **Dipak Nayak:** Investigation, Funding acquisition, Formal analysis, Data curation. **Ashok Yadav:** Funding acquisition, Formal analysis, Data curation. **Tilak Mondal:** Methodology, Investigation, Formal analysis, Data curation. **Rakesh Kr Ghosh:** Methodology, Funding acquisition, Formal analysis, Data curation. **Manik Bhowmick:** Methodology, Investigation, Formal analysis, Data curation. **Atul Singha:** Methodology, Investigation, Funding acquisition, Formal analysis, Data curation. **D.P. Ray:** Formal analysis, Data curation. **B.S. Manjunatha:** Methodology, Investigation, Formal analysis, Data curation. **Avijit Das:** Methodology, Investigation, Funding acquisition, Formal analysis, Data curation. **D.B. Shakyawar:** Investigation, Funding acquisition, Formal analysis, Data curation. **Sourav Paul:** Methodology, Investigation, Formal analysis, Data curation. **Amit Das:** Formal analysis, Data curation, Conceptualization. **Santanu Mukherjee:** Writing – review & editing, Supervision. **Ravinder Kumar:** Writing – review & editing, Supervision, Funding acquisition, Formal analysis.

## Ethical compliance

We have meticulously followed the Guide for Authors to prepare this manuscript, ensuring compliance with the Ethics in Publishing Policy outlined in the Guide for Authors.

## Disclosure statement

The authors declare the following financial interests/personal relationships which may be considered as potential competing interests:

Ravinder Kumar is working as an associate editor in the Journal.

## Data availability statement

The datasets generated and/or analyzed during the current study are available within the article. The raw data can be made available on request.

## Funding

No funding was available for publishing the current study.

## Declaration of competing interest

The authors declare the following financial interests/personal relationships which may be considered as potential competing interestsThe authors declare the following financial interests/personal relationships which may be considered as potential competing interests: Ravinder Kumar is working as an associate editor in the Journal.

If there are other authors, they declare that they have no known competing financial interests or personal relationships that could have appeared to influence the work reported in this paper.

## References

[1] Chakraborty D., Nagarajan S., Aggarwal P., Gupta V.K., Tomar R.K., Garg R.N., Sahoo R.N., Sarkar A., Chopra U.K., Sarma K.S.S., Kalra N. (2008). Effect of mulching on soil and plant water status, and the growth and yield of wheat (Triticum aestivum L.) in a semi-arid environment. Agric. Water Manag..

[2] Machado A.A.S., Lau C.W., Till J., Kloas W., Lehmann A., Becker R., Rillig M.C. (2018). Impacts of microplastics on the soil biophysical environment. Environ. Sci. Technol..

[3] Amare G., Desta B. (2021). Coloured plastic mulches: impact on soil properties and crop productivity. Chem. Biol. Technol. Agric..

[4] Kirigiah R., Peter M., Erick M.G. (2022). Effect of plastic mulch color and transplanting stage on baby corn plant performance. Eur. J. Agric. Food Sci..

[5] Sánchez-Hernández J.C., Capowiez Y., Ro K.S. (2020). Potential use of earthworms to enhance decaying of biodegradable plastics. ACS Sustain. Chem. Eng..

[6] Filipović V., Bristow K.L., Filipović L., Wang Y., Sintim H.Y., Flury M., Šimůnek J. (2020). Sprayable biodegradable polymer membrane technology for cropping systems: challenges and opportunities. Environ. Sci. Technol..

[7] Han Y., Wei M., Han F., Fang C., Wei D., Zhong Y., Li F. (2020). Greater biofilm formation and increased biodegradation of polyethylene film by a microbial consortium of Arthrobacter sp. and Streptomyces sp. Microorganisms.

[8] Ding F., Li S., Lu J., Penn C.J., Wang Q., Lin G., Rillig M.C. (2023). Consequences of 33 years of plastic film mulching and nitrogen fertilization on maize growth and soil quality. Environ. Sci. Technol..

[9] Zhang J., Zou G., Wang X., Ding W., Xu L., Liu B., Chen Y. (2021). Exploring the occurrence characteristics of microplastics in typical maize farmland soils with long-term plastic film mulching in northern China. Front. Mar. Sci..

[10] Derraik J.G.B. (2002). The pollution of the marine environment by plastic debris: a review. Mar. Pollut. Bull..

[11] Shimao M. (2001). Biodegradation of plastics. Curr. Opin. Biotechnol..

[12] Maity S., Singha K., Gon D.P., Paul P., Singha M. (2012). A review on jute non-wovens: manufacturing, properties and applications. Int. J. Textile Sci..

[13] Maity S., Gon D.P., Paul P. (2014). A review of Flax Non-wovens: manufacturing, properties and applications. J. Nat. Fibers.

[14] Faraca G., Astrup T. (2019). Plastic waste from recycling centres: characterisation and evaluation of plastic recyclability. Waste Manag..

[15] Eriksen M.K. (2018). Contamination in plastic recycling: influence of metals on the quality of reprocessed plastic. Waste Manag..

[16] Law D.M., Rowell A.B., Snyder J.C., Williams M.A. (2006). Weed control efficacy of organic mulches in two organically managed bell pepper production systems. HortTechnology.

[17] Bond W., Grundy A.C. (2001). Non‐chemical weed management in organic farming systems. Weed Res..

[18] Barman D. (2022). Net ecosystem CO2 exchange from jute crop (Corchorus olitorius L.) and its environmental drivers in tropical Indo-Gangetic plain using open-path eddy covariance technique. Environ. Monit. Assess..

[19] FAOSTAT (2018). FAOSTAT statistical database. http://www.fao.org/faostat/en/#data/QC.

[20] (2020). Directorate of Economics and Statistics, Ministry of Agriculture, Government of India, dacnet.nic.in.

[21] Noyon A.R., Karim S., Rouf A., Jamal M., Layek R.K., Sivanantham G., Uddin E. (2023). Fabrication of biodegradable kraft paper from buffing dust and jute fiber: green solutions for packaging. Polym. Eng. Sci..

[22] Mridha N., Ray D.P., Saha B., Ghosh R.K., Das A., Bhowmick M., Shakyawar D.B. (2022). Natural fibre based non-woven agrotextile mulch: a boon for natural farming. Indian Farming.

[23] Ganguly P.K., Bhaduri S.K., Day A. (2004).

[24] Saha B., Prasad L.K., Harris A., Sikka A.K., Batta R.A. (2006). Effect of geo-textile mulch on soil moisture, temperature and yield of vegetable crops grown in planes of Bihar. Int. J. Trop. Agric..

[25] Nag D., Choudhury T.K., Debnath S., Ganguly P.K., Ghosh S.K. (2008). Efficient management of soil moisture with jute non-woven as mulch for cultivation of sweet lime and turmeric in red lateritic zone. J. Agric. Eng..

[26] Manna K., Kundu M.C., Saha B., Ghosh G.K. (2018). Effect of nonwoven jute agrotextile mulch on soil health and productivity of broccoli (Brassica oleracea L.) in lateritic soil. Environ. Monit. Assess..

[27] Das S.P., Bera M., Sen J., Ghosh G.K., Saha B., Debnath S., Roy S.B., Das D., Mondal S., Biswas P.K., Kundu M.C. (2017). Efficacy of geotextile jute mulches on yield, soil nutrient dynamics and weed suppression in French bean (Phaseolus vulgaris L.)–Capsicum (Capsicum annum L.) cropping system. Int. J. Bio-resource Stress Manag..

[28] Kumar P., Jaslam P.K.M., Chandanshive A. (2022). Modelling and forecasting of area, production and productivity of tomatoes in Haryana and India. Indian J. Ext. Educ..

[29] Malik Z.A., Lal E.P., Mir Z.A., Lone A.H. (2019). Effect of salicylic acid and indole acetic acid on tomato crop under induced salinity and cadmium-stressed environment: a review. Int. J. Plant Soil Sci..

[30] Sękara A., Pokluda R., Cozzolino E., Piano L.d., Cuciniello A., Caruso G. (2019). Plant growth, yield, and fruit quality of tomato affected by biodegradable and non-degradable mulches. Hortic. Sci..

[31] Anzalone A., Cirujeda A., Aibar J., Pardo G., Zaragoza C. (2010). Effect of biodegradable mulch materials on weed control in processing tomatoes. Weed Technol..

[32] TAPPI Standard and suggested methods (1971).

[33] Sarkar P.B., Mazumdar A.K., Pal K.B. (1948). The hemicelluloses of jute fibre. J. Textil. Inst..

[34] Dasgupta P.C., Sardar D., Mazumder A.K. (1976). Chemical retting of jute. Food Farming Agric.

[35] ASTM International (2023).

[36] Hearle J.W.S., Sultan M.A. (1967). The use of tensile tests for the prediction of fabric properties. J. Textile Inst. Trans..

[37] ASTM International (2023).

[38] ASTM International (2023).

[39] British Standards Institution (BSI) (2005).

[40] Bureau of Indian Standards (BIS) (1992).

[41] Schmugge T.J., Jackson T.J., McKim H.L. (1980). Survey of methods for soil moisture determination. Water Resour. Res..

[42] Twarakavi N.K.C., Šimůnek J., Schaap M.G. (2010). Can texture‐based classification optimally classify soils with respect to soil hydraulics?. Water Resour. Res..

[43] Faé G.S., Montes F., Bazilevskaya E., Añó R.M., Kemanian A.R. (2019). Making soil particle size analysis by laser diffraction compatible with standard soil texture determination methods. Soil Sci. Soc. Am. J..

[44] Jackson M.L. (1973).

[45] Walkley A., Black I.A. (1934). An examination of Degtjareff method for determining soil organic matter, and a proposed modification of the chromic acid titration method. Soil Sci..

[46] Subbaiah B.V., Asija G.L. (1956). A rapid procedure for the estimation of available nitrogen in soil. Curr. Sci..

[47] Olsen S.R., Cole C.V., Watanabe F.S., Dean L.A. (1954).

[48] Klípa V., Šimůnek J., Šejna M., Snehota M. (2015). Estimation of unsaturated hydraulic conductivity using a minidisk infiltrometer and numerical inversion. J. Hydrol. Hydromechanics.

[49] Lobsey C., Rossel R.A.V. (2016). Sensing of soil bulk density for more accurate carbon accounting. Eur. J. Soil Sci..

[50] Aechra S., Meena R.H., Meena S., Mundra S., Lakhawat S.S., Mordia A., Jat G. (2021). Soil microbial dynamics and enzyme activities as influenced by biofertilizers and split application of vermicompost in rhizosphere of wheat (Triticum aestivum L.). J. Environ. Biol..

[51] Singh N.B., Hamimed S. (2022). Soil chemical properties, microbial biomass, and soil enzyme dynamics on transplanted rice with organic amendments. Res. Sq.

[52] Bremner J.M., Mulvaney C.S. (1982). Methods of Soil Analysis, Part 2: Chemical and Microbiological Properties.

[53] Singleton V.L., Rossi J.A. (1965). Colorimetry of total phenolics with phosphomolybdic-phosphotungstic acid reagents. Am. J. Enol. Vitic..

[54] Zhishen J., Mengcheng T., Jianming W. (1999). The determination of flavonoid contents in mulberry and their scavenging effects on superoxide radicals. Food Chem..

[55] Imran A., Sardar F., Khaliq Z., Nawaz M.S., Shehzad A., Ahmad M.S.A., Mirza M.N. (2022). Tailored bioactive compost from agri-waste improves the growth and yield of chili pepper and tomato. Front. Bioeng. Biotechnol..

[56] McCullough D.R., Wilson K.R. (2002).

[57] Geminiani L., Campione F.P., Corti C., Luraschi M., Motella S., Recchia S., Rampazzi L. (2022). Differentiating between natural and modified cellulosic fibres using ATR-FTIR spectroscopy. Heritage.

[58] Abidi H., Rana S., Chaouch W., Azouz B., Aïssa I.B., Hassen M.B., Fangueiro R. (2019). Accelerated weathering of textile waste nonwovens used as sustainable agricultural mulching. J. Ind. Text..

[59] Marasović P., Kopitar D., Brunšek R., Schwarz I. (2023). Performance and degradation of non-woven mulches made of natural fibres and PLA polymer—open field study. Polymers.

[60] Xu J., Zhang Y., Wang W. (2016). Organic mulches improve microclimate and soil moisture in cropland: a global meta-analysis. Agric. For. Meteorol..

[61] Noor M., Ullah A., Al-Sadi A.M., Mabood F. (2020). Impact of straw mulch on soil temperature, moisture, and maize growth under semiarid conditions. J. Soil Sci. Plant Nutr..

[62] Ramakrishna A., Tam H.M., Wani S.P., Long T.D. (2006). Effect of mulch on soil temperature, moisture, weed infestation and yield of groundnut in northern Vietnam. Field Crops Res..

[63] Jordan A., Zawala L.M., Gill J. (2010). Effects of mulching on soil physical properties and runoff under semi-arid conditions in southern Spain. Catena.

[64] Bakshi P., Wali V.K., Iqbal M., Jasrotia A., Kour K., Ahmed R., Bakshi M. (2015). Sustainable fruit production by soil moisture conservation with different mulches: a review. Afr. J. Agric. Res..

[65] Peng-fei Z., Zhang Z., Xiao M., Chao J., Dai Y., Li G., Senge M. (2023). Effects of organic mulching on moisture and temperature of soil in greenhouse production of tomato under unheated greenhouse cultivation in the cold zone of China. Food Sci. Nutr..

[66] O E., Rocha A.M., Catarino A. (2011). The influence of knitted fabrics' structure on the thermal and moisture management properties. J. Eng. Fibers Fabr..

[67] Subba R. (2015).

[68] Wang X., Chen H., Liu J., Zhang Y. (2021). Mulching enhances soil microbial community structure and carbon retention in agricultural ecosystems. Soil Biol. Biochem..

[69] Liu Z. (2023). Influence of mulching practices on soil bacterial communities and carbon dynamics. Agric. Res..

[70] Gopalakrishnan S., Watanabe T., Pearse S.J., Ito O., Hossain Z.A.K.M., Subbarao G.V. (2009). Biological nitrification inhibition by Brachiaria species: a novel strategy to improve nitrogen use efficiency in cropping systems. Plant Soil.

[71] Yadav A., Yadav K. (2013). Seasonal population dynamics of rhizosphere and non-rhizosphere soil microorganism of chirpine seedlings (Pinus roxburghiiSarg.). Br. Microbiol. Res. J..

[72] Pal D., Bera S., Ghosh R.K. (2013). Influence of herbicides on soybean yield, soil microflora and urease enzyme activity. Indian J. Weed Sci..

[73] Wilen C.A., Schuch U.K., Elmore C.L. (1999). Mulches and subirrigation control weeds in container production. J. Environ. Hortic..

[74] Shehata S.A., Abdelhamid M.T., Abou El-Yazied A.A. (2019). Effect of mulching on growth, yield, and nutrient uptake of tomato plants under arid conditions. J. Plant Nutr..

[75] Odediran A., Yu J., Gu S. (2023). The effect of layers of high tunnel covering and soil mulching on tomato fruit quality. J. Sci. Food Agric..

